# Oral Administration of Quercetin or Its Derivatives Inhibit Bone Loss in Animal Model of Osteoporosis

**DOI:** 10.1155/2020/6080597

**Published:** 2020-10-27

**Authors:** Yue-Yue Huang, Zi-Hao Wang, Li-Hui Deng, Hong Wang, Qun Zheng

**Affiliations:** ^1^Department of Hematology, The Second Affiliated Hospital and Yuying Children's Hospital of Wenzhou Medical University, Wenzhou, China; ^2^Department of Rheumatology Immunology, The Second Affiliated Hospital and Yuying Children's Hospital of Wenzhou Medical University, Wenzhou, China

## Abstract

*Objectives*. *Quercetin* (Q) and its derivatives are the major members of the naturally occurring flavonoid family, which possess beneficial effects on disease prevention including osteoporosis. The present study is aimed at further investigating the efficacy of the Q and its derivatives on bone pathology, bone-related parameters under imageology, bone maximum load, and serum bone metabolism indexes in animal model of osteoporosis. Potential mechanisms of Q and its derivatives in the treatment of osteoporosis as well as the existing problems regarding the modeling method and limitations of researches in this area were also summarized. Eight databases were searched from their inception dates to February 2020. Nineteen eligible studies containing 21 comparisons were identified ultimately. The risk of bias and data on outcome measures were analyzed by the CAMARADES 10-item checklist and Rev-Man 5.3 software separately. The results displayed the number of criteria met varied from 3/10 to 7/10 with an average of 5.05. The present study provided the preliminary preclinical evidence that oral administration of Q or its derivatives was capable of improving bone pathology, bone-related parameters under imageology and bone maximum load, increasing serum osteocalcin, alkaline phosphatase, and estradiol, and reducing serum c-terminal cross-linked telopeptide of type I collagen (*P* < 0.05). No statistical difference was seen in survival rate, index of liver, or kidney function (*P* > 0.05). Q and its derivatives partially reverse osteopenia probably via antioxidant, anti-inflammatory, promoting osteogenesis, inhibiting osteoclasts, and its estrogen-like effect. The findings reveal the possibility of developing Q or its derivatives as a drug or an ingredient in diet for clinical treatment of osteoporosis.

## 1. Introduction

Osteoporosis, as a systemic skeletal disease characterized by loss of bone mass and bone microarchitectural deterioration, causes enhanced bone fragility and a greater risk of fractures [1]. Some risk factors are bound up with osteopathic fracture, containing age, race, cigarette smoking, low physical activity, hormone-related factors, the use of drugs (e.g., glucocorticoids), low calcium and vitamin D levels, and prior history of fracture [2]. It gradually becomes a major public health issue affecting exceeded 200 million people worldwide [3], and the burden of society is continuously increasing with the aging of the world population [4]. Thus, how to effectively prevent and treat osteoporosis has attracted more and more attention of the world.

Physical activity [5], supplementing with calcium and vitamin D, [6, 7] and pharmacological therapies [8] (including estrogen [9], bisphosphonates [10], denosumab [11], or teriparatide [12]) are the most commonly used clinical approaches for the prevention or treatment of osteoporosis. However, there is no definite solution for this disease because there are still some problems in the above treatment methods [13]. For example, although bisphosphonates are widely used globally as the main treatment for osteoporosis, it does not include proven efficacy against nonvertebral fractures, and the evidence for benefit beyond 5 years in intervention studies is limited [14]. Besides, there are concerns that atypical femur fractures and osteonecrosis of the jaw may be caused by long-term use of bisphosphonates [14]. High-impact exercise (e.g., running) is conducive to producing changes that reflect distal tibial bone adaptation [5]. However, a well-designed training intervention is difficult to achieve in normal people much less in patients with osteoporosis or fracture. The benefits of supplementing with vitamin D and calcium for fracture prevention and holistic fall remain uncertain [15]. There are inconsistent findings for adverse effects on the cardiovascular system of calcium supplements with or without vitamin D [7, 16, 17], and the adverse effects on the skeleton and musculature of high-dose vitamin D were reported by recent evidence [18, 19]. Despite its excellent antiosteoporosis effect for postmenopausal osteoporosis, long-term use of estrogen for osteoporosis caused many side effects, containing an increased incidence of cardiovascular accidents, endometrial cancer [20–22], as well as the loss of mesenteric ganglion neurons and dominant ovarian follicles [23]. Therefore, obtaining a new effective drug without side effects is needed urgently in the treatment of osteoporosis.

**Figure 1:**
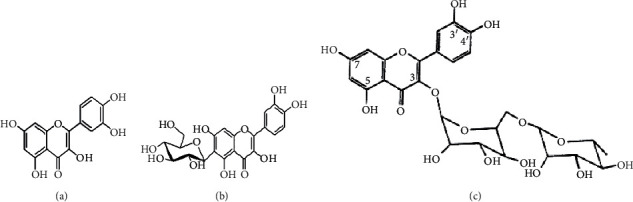
The chemical structure of Q and its derivatives.

Quercetin (Q, C_15_H_10_O_7_, Figure 1(a)) and its derivatives (Figures 1(b) and 1(c)) are the major members of the naturally occurring flavonoid family. Flavonoid is well known for its antioxidant and anti-inflammatory properties. Q and its derivatives have been widely used as a natural antioxidant in the treatment of cardiovascular diseases, tumor, and some metabolic diseases including osteoporosis for many years in China [24, 25]. Recent studies [26, 27] demonstrate that Q and its derivatives are potent natural osteogenic agents by multiple pharmacological activities including the function of antioxidant, anti-inflammatory, and estrogen-like effect *in vivo* and *vitro* studies. However, the scattered evidence and uncertain mechanisms limited the application of Q and its derivatives in the clinic. Systematic review and meta-analysis of animal studies are considered to be a valuable tool to provide important insights into the validity of animal studies, improve the precision of estimated effects, and support further generalization to human clinical trials [28]. Thus, the present study is aimed at investigating the preclinical evidence and possible mechanisms of Q and its derivatives in animal models of osteoporosis.

## 2. Methods

### 2.1. Data Sources and Search Strategies

A comprehensive literature search about animal experimental studies of Q or its derivatives for osteoporosis was conducted in the Chinese Science and Technology Journal Database, WanFang, China National Knowledge Infrastructure, Chinese Biomedical Database, PubMed, EMBASE, Cochrane library, and Web of Science database from their inception dates to February 2020. The following search terms were used in PubMed and were modified to suit other databases: “Quercetin” AND “Osteoporosis OR Bone Loss”. In addition, reference lists from the resulting publications and reviews were also searched carefully for the eligible studies.

### 2.2. Eligibility Criteria and Data Extraction

Two authors (Yue-Yue Huang and Zi-Hao Wang) selected the studies separately by browsing the abstracts and full texts via the eligibility criteria. The study was included if it met the following criteria: (1) controlled studies assessing the administration of Q and its derivatives for osteoporosis or bone loss animal models established by various ways; (2) the treatment group received Q or its derivatives as monotherapy with unrestricted dosage, medicament type, route of administration, and time for the medicine application. Blank treatment or isasteric placebo was received in the control group; (3) the present study received bone pathology and/or bone mineral density (including femur bone mineral density (F-BMD), lumbar spine bone mineral density (L-BMD)) and/or bone histomorphometric parameters under micro-CT (trabecular number (Tb.N), trabecular thickness (Tb.Th)) and/or bone maximum load and/or bone turnover markers (serum alkaline phosphatase (ALP), C-terminal cross-linked telopeptide of type I collagen (CTX) and osteocalcin (OC)) and/or serum estradiol and/or uterine weight and/or indicators of adverse reactions as the primary outcome measures, while the antiosteoporosis mechanisms of Q or its derivatives was selected as the second outcome measures. Exclusion criteria was as below: (1) other types of studies (*in vitro* studies, case reports, clinical trials, reviews, abstracts, comments, and editorials); (2) combination with other compounds; (3) compared with other traditional Chinese medicine; (4) no any primary outcome indicator were involved or incomplete date; (5) inconsistent of graphic and textual data; (6) no control group; (7) duplicate publications; (8) not osteoporosis or bone loss model.

The details were extracted from included studies by two independent authors (Yue-Yue Huang and Zi-Hao Wang) using a predefined form. The information included the authors and years of publication; information of animals; modeling method; the use of anesthetics anaesthetize in the course of the experiment; the therapeutic regimen of treatment and control group; and primary and/or secondary outcomes and its intergroup differences. Only data from the osteoporosis group and Q+osteoporosis groups were included for analysis when a study is involved in multiple intervention groups. When the outcomes were displayed through gradient doses of drug therapy or determined at different times, only the data of the highest dose and the final measurement was included for analysis.

### 2.3. Risk of Bias in Individual Studies

CAMARADES 10-item quality checklist [29] with minor modification was used to assess the study quality by two independent authors (Yue-Yue Huang and Li-Hui Deng). The modification is listed as follows: D: blinded induction of model (group randomly after modeling); F: use of anesthetic without significant bony protective activity or nephrotoxicity; G: appropriate animal model with complications or risk factors (including age, hyperlipemia, diabetes, or hypertensive). Disagreements in the process of selecting studies, extracting data, and assessing the quality of studies were resolved by consensus or arbitration by the correspondence author (Qun Zheng).

### 2.4. Statistical Analysis

The RevMan 5.3 software was used for data analysis where possible; otherwise, comparison between groups was performed. The bar graphs were drawn via Prism 6. In meta-analysis, standardized mean differences (SMDs) and 95% confidence intervals (95% CIs) were calculated to estimate the combined overall effect sizes when the outcomes were determined in various ways or the unit of measurement is different. Heterogeneity was assessed using the Cochrane Q-statistic test (*P* < 0.05 was considered statistically significant) and the *I*^2^-statistic test (*I*^2^ < 50% was considered homogeneous). Random (*I*^2^ > 50%) or fixed-effects model (*I*^2^ < 50%) was selected according to the results of *I*^2^. In order to ensure the reliability of results, a sensitivity analysis was performed, and potential publication bias was assessed by the visual inspection of the funnel plot and asymmetry test. Moreover, in order to explore the impact of potential confounding factors on the estimates of combined effect size, subgroup analyses were conducted in this study. The significance level was set at *P* < 0.05.

## 3. Results

### 3.1. Study Selection

**Figure 2:**
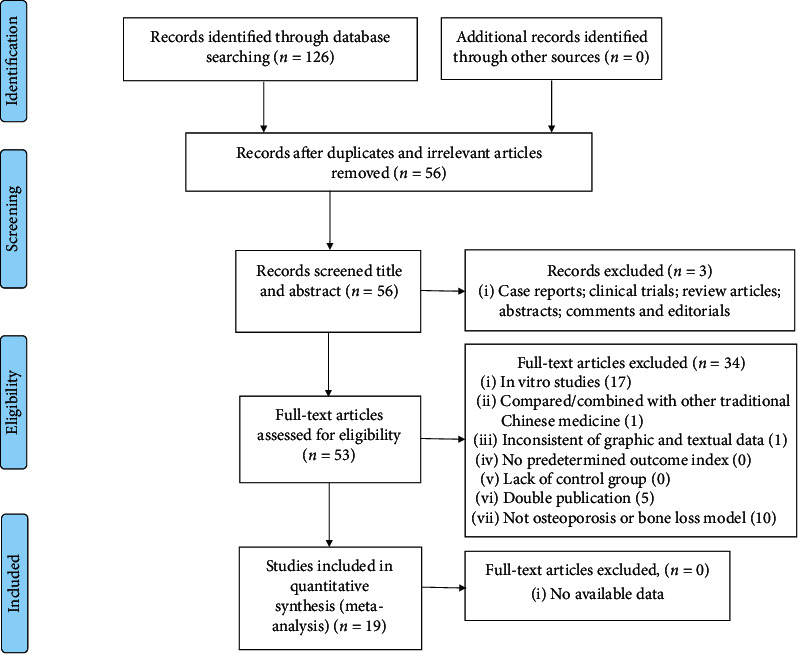
Summary of the process for identifying candidate studies.

A total of 126 studies were identified by the initial database search and 19 eligible studies [4, 27, 30–46] containing 21 comparisons were included in this study ultimately. The process followed for study selection is shown in Figure 2.

### 3.2. Characteristics of Included Studies

The detailed characteristics of the included studies were generalized in Table 1. Nine English studies and 8 Chinese studies between 2000 and 2019 with 414 animals were identified. The sample size of each study ranged from 14 to 40 animals. Female Sprague-Dawley (SD) rats (51.5%), female Wistar rats (9.42%), female Y59 rats (4.7%), female C57BL/6 mice (15.6%), male SD rats (4.7%), male Wistar rats (4.7%), and male C57BL/6 mice (9.42%) were used in the studies. The weight of SD, Wistar, and Y59 rats varied between 170 g and 350 g, and the weight of mice varied between 16.5 g and 18.8 g. Fourteen [4, 27, 30, 32–35, 37, 38, 43–46] studies established osteoporosis or bone loss model by bilateral oophorectomy; 2 studies [39, 40] by feeding with high-fat diet for several weeks, 1 study [42] by intraperitoneal injection of STZ (100 mg/kg), 1 study [31] by oral gavage of isotretinoin (80 mg/kg, qd) for 14 days, 1 study [41] by subcutaneous injection of methylprednisolone sodium succinate (40 mg/kg body mass) for 6 weeks, and 1 study [36] by oral gavage of n-ZnO (600 mg/kg, qd) for 5 consecutive days. Anesthetics were reported in 13 studies. Of which, chloral hydrate was reported in 4 studies [4, 35, 37, 46], mixture of ketamine and xylazine in 2 studies [31, 41], ketamine in 2 studies [34, 44], sodium pentobarbital in 2 studies [30, 38], ether in 1 study [36], ethyl ether in 1 study [39], and diethyl ether in 1 study [42]. Detailed information of Q or its derivatives in each study is displayed in Table 2. All studies implemented different doses of Q or its derivatives by oral or intragastric administration. Among them, 14 studies [27, 30–38, 41, 42, 44, 45] reported the dose gradient of Q ranged 5 mg/kg/d to 300 mg/kg/d, 2 studies [39, 40] utilized a standard high-fat diet plus 0.01% Q per day, 1 study [43] utilized a standard high-fat diet plus 2.5% Q (5 g per mouse) per day, 1 study [27] utilized quercetin-6-C-A-D-glucopyranoside (QCG) with 5 mg/kg/d, and 2 studies [4, 46] utilized quercetin-3-O-rutinose with 2.5 mg/kg/d. Bone pathology was utilized as primary outcome measure in 3 studies [31, 38, 41], F-BMD in 13 studies [4, 27, 30–34, 39, 42–46], L-BMD in 3 studies [35, 43, 44], Tb.Th in 4 studies [27, 30, 33, 43], Tb.N in 4 studies [27, 30, 33, 43], bone maximum load in 9 studies [27, 32, 34, 37, 39–42, 45], serum ALP in 8 studies [30, 31, 33–36, 42, 45], serum OC in 4 studies [35, 37, 41, 42], CTX in 3 studies [34, 35, 41], survival rate in 4 studies [4, 33, 38, 45], blood urea nitrogen (BUN) in 2 studies [31, 34], serum creatinine (SCr) in 1 study [34], aspartate aminotransferase (AST) in 1 study [31], and alanine aminotransferase (ALT) in 1 study [31]. Serum Superoxide dismutase (SOD) was reported as second outcome measure in 3 studies [31, 39, 42]; serum catalase (CAT) in 2 studies [31, 42]; serum malondialdehyde (MDA) in 3 studies [31, 39, 40]; serum glutathione peroxidase (GSH) in 4 studies [31, 39, 40, 42]; serum estradiol in 3 studies [35, 37, 45]; uterine weight in 3 studies [27, 33, 43]; serum tumor necrosis factor-*α* (TNF-*α*) in 3 studies [4, 32, 36]; interleukin-6 in 2 studies [4, 36]; interferon *γ* (INF-*γ*) in 1 study [4]; C-reactive protein (CRP) in 1 study [36]; nuclear factor-*κ* B (NF-*κ*B) in 1 study [32]; serum NO in 1 study [36]; serum Ca and P in 4 studies [30, 35, 41, 46]; urinary Ca and P in 3 studies [30, 33, 35, 46]; haematological parameters in 1 study [31]; extracellular regulated protein kinases (ERK), amino-terminal protein kinas (JNK), and P38 in 1 study [34]; bone morphogenetic protein 2 (BMP2); and smad family member 4 (Smad4) in 1 study [35]; serum col1a1, bone Gla protein 2 (Bglap2), NF-E2-related factor 2 (Nrf-2), thyroid hormone receptor *α*1 (TR*α*1), and glycogen synthase kinase 3*β* (GSK-3) in 1 study [39]; cathepsin K (CTSK) in 2 studies [39, 43]; receptor activator of nuclear factor-*κ* B (RANK) in 2 studies [27, 39]; Runt-related transcription factor 2 (Runx2) in 1 study [30]; Forkhead box transcription factor O1 (FoxO1) in 1 study [30]; macrophage colony-stimulating factor (M-CSF) and c-fos in 1 study [27]; calcitonin receptor (CTR), matrix metalloproteinase 9 (MMP9); and nuclear factor of activated T cells c1 (NFATc1) in 1 study [43].

### 3.3. Study Quality

Detailed results of methodological quality are presented in Table 3. The number of criteria met in each study varied from 3/10 to 7/10 with the average of 5.05. Only 2 studies [30, 39] were not a peer-reviewed publication, and 3 studies [32, 44, 45] did not mention control of temperature. Two [31, 46] of the included studies did not declare randomization. The ways of blinding induction of model were reported in 5 studies [4, 31, 35, 36, 43], and all of them reported the animals were grouped randomly after modeling. No study mentioned the calculation of sample size and none used a blinding method during outcome assessment and appropriate animal model. Thirteen studies [4, 30, 31, 34–39, 41, 42, 44, 46] used the anesthetic without protective and toxic effects on bones. Compliance with animal welfare regulations was not described in 3 studies [27, 38, 42,], and the potential conflict of interests was not mentioned in 7 studies [4, 30, 34, 39, 44–46].

### 3.4. Effectiveness

#### 3.4.1. Bone Pathology

Three studies [31, 38, 41] utilized bone pathology as a primary outcome measure. Among them, 1 study [31] reported that osteoporotic rats treated with Q showed marked the improvement of the structure of femoral cortical bone compared with osteoporotic rats induced by 13cRA, which showed thickness was nearly similar to that of the control group although a few small intracortical cavities were still present. One study [38] reported that Q treatment was observed to prevent trabecular fracture and osteoblast apoptosis and maintain normal distribution of trabecular. Another study [41] reported that the administration of 150 mg/kg Q increased femoral trabecular and cortical thickness by 36% and 22%, respectively, compared with the osteoporotic rats induced by methylprednisolone sodium succinate.

#### 3.4.2. Bone Related Parameters under Imageology and Bone Maximum Load

**Figure 3:**
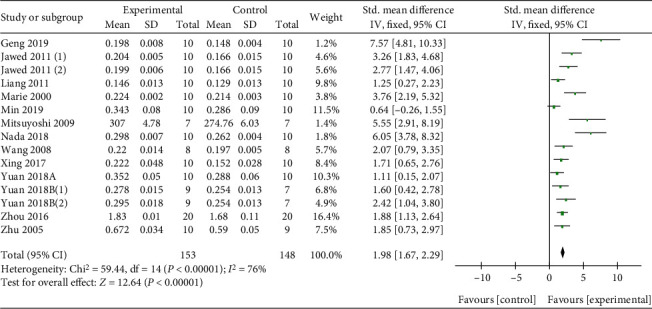
The forest plot: effects of Q or its derivatives for increasing L-BMD compared with the control group.

**Figure 4:**
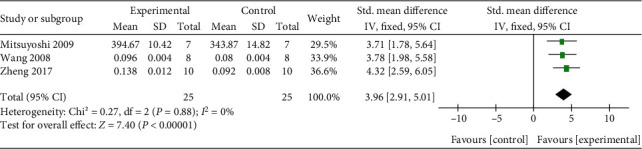
The forest plot: effects of Q or its derivatives for increasing F-BMD compared with the control group.

**Figure 5:**
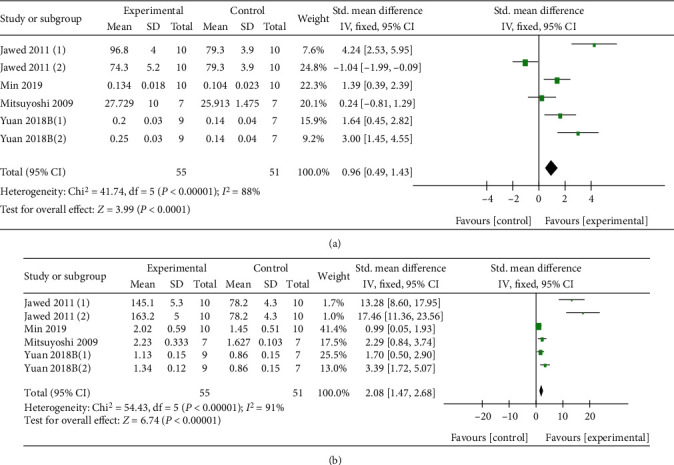
(a) The forest plot: effects of Q or its derivatives for increasing Tb.Th compared with the control group; (b) The forest plot: effects of Q or its derivatives for increasing Tb.N compared with the control group.

**Figure 6:**
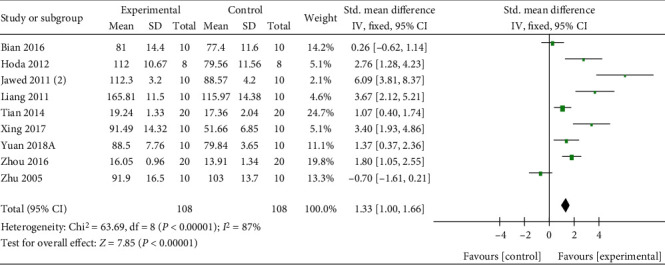
The forest plot: effects of Q or its derivatives for increasing bone maximum load compared with the control group.

With dual-energy X-ray absorptiometry, meta-analysis of 15 researches [27, 31–34, 39, 42–46] and 3 researches [4, 30, 35, 43, 44] separately showed a significant effect of Q or its derivatives for increasing F-BMD (*n* = 301, SMD 1.98, 95% CI (1.67, 2.29), *P* < 0.00001; heterogeneity: *χ*^2^ = 59.44, *I*^2^ = 76%, Figure 3) and L-BMD (*n* = 50, SMD 3.96, 95% CI (2.91, 5.01), *P* < 0.00001; heterogeneity: *χ*^2^ = 0.27, *I*^2^ = 0%, Figure 4) compared with the control group. Under micro-CT, meta-analysis of 6 researches [27, 30, 33, 43] and 6 researches [27, 30, 33, 43] separately showed a significant effect of Q or its derivatives for increasing Tb.Th (*n* = 106, SMD 0.96, 95% CI (0.49, 1.43), *P* < 0.00001; heterogeneity: *χ*^2^ = 41.74, *I*^2^ = 88%, Figure 5(a)) and Tb.N (*n* = 106, SMD 2.08, 95% CI (1.47, 2.68), *P* < 0.00001; heterogeneity: *χ*^2^ = 54.43, *I*^2^ = 91%, Figure 5(b)). About physical mechanics index, meta-analysis of 9 researches [27, 32, 34, 37, 39–42, 45] showed a significant effect of Q or its derivatives for increasing bone maximum load (*n* = 216, SMD 1.33, 95% CI (1.0, 1.66), *P* < 0.00001; heterogeneity: *χ*^2^ = 63.69, *I*^2^ = 87%, Figure 6). In consideration of high heterogeneity, sensitivity analyses of the above indicators were carried out, and the result showed that the heterogeneity did not substantially alter after removing any 1 study.

#### 3.4.3. Serum ALP, OC, and CTX

Compared with the control group, Q and its derivatives were reported that they existed significant effect for increasing serum ALP (*P* < 0.05) in 7 studies [30, 33–36, 42], no significant effect for serum ALP (*P* > 0.05) in 1 study [31], and significant effect for reducing serum ALP (*P* < 0.05) in 1 study [45]. Besides, 5 studies [30, 35, 37, 41, 42] and 3 studies [34, 35, 41] reported separately that Q could increase the serum OC (*P* < 0.05) and/or reduce the serum CTX (*P* < 0.05).

#### 3.4.4. Serum Estradiol and Uterine Weight

**Figure 7:**
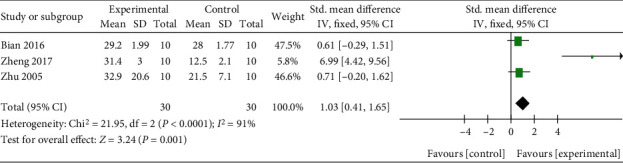
The forest plot: effects of Q or its derivatives for increasing serum estradiol level compared with the control group.

**Figure 8:**
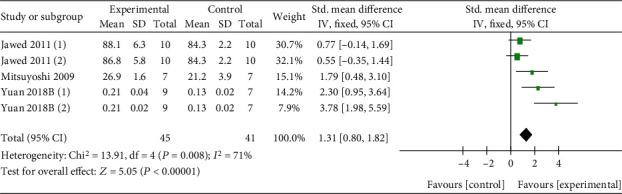
The forest plot: effects of Q or its derivatives for increasing uterine weight of experimental animals compared with the control group.

Q and its derivatives were found to increase serum estradiol level relatively (*n* = 60, SMD 1.03, 95% CI (0.41, 1.65), *P* < 0.00001; heterogeneity: *χ*^2^ = 21.95, *I*^2^ = 91%, Figure 7). As a result of estradiol effects, the uterine weight of experimental animals was slightly increased compared with the control group (*n* = 86, SMD 1.31, 95% CI (0.80, 1.82), *P* < 0.00001; heterogeneity: *χ*^2^ = 13.91, *I*^2^ = 71%, Figure 8).

#### 3.4.5. Indicators of Adverse Reactions

**Figure 9:**
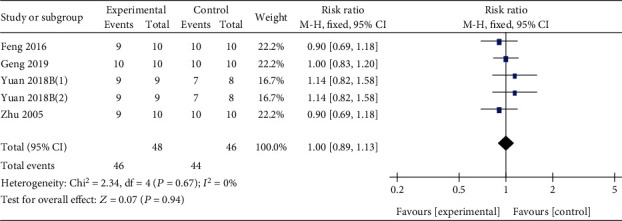
The forest plot: effects of Q or its derivatives on the survival rate of experimental animals compared with the control group.

Four studies [4, 33, 38, 45] utilized survival rate as primary outcome measure, and meta-analysis of 3 studies showed no statistical difference of Q or its derivatives on survival rate (*n* = 94, RR 1.00, 95% CI (0.89, 1.13), *P* = 0.94; heterogeneity: *χ*^2^ = 2.34, *I*^2^ = 0%, Figure 9). BUN was measured in 2 studies [31, 34] and SCr in 1 study [34] to assess the adverse effect of Q to the renal function. In addition, AST and ALT were measured in 1 study [31] to assess the adverse effect of Q to the liver, and the results showed that there was no statistical difference in renal and liver function between Q group and control group (*P* > 0.05).

### 3.5. Subgroup Analysis

**Figure 10:**
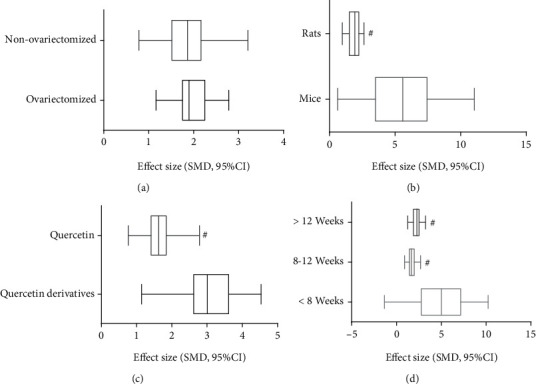
Subgroup analyses of the F-BMD. (a) The different effect size between the ovariectomized model group and nonovariectomized model group; (b) the different effect size between mice and rats; (c) the different effect size between Q and its derivatives; (d) the different effect size between different treatment time group. ^#^*P* < 0.05 vs. control groups; ^∗^*P* > 0.05 vs. control groups.

F-BMD was reported to be improved greatly in 15 comparisons [4, 27, 30–34, 39, 42–46]. The potential confounding factors which may increase the heterogeneity of F-BMD were explored via subgroup analysis. First, we divide the 15 comparisons into the ovariectomized model group and nonovariectomized model group according to different modeling methods. As the result, no significant difference was observed in the effect size of two groups (SMD = 2.00 ± 0.36 versus SMD = 1.93 ± 0.58, *P* = 0.85, Figure 10(a)) and heterogeneity of both groups did not decrease obviously. On the basis of the result of the subgroup analysis above, we analyzed the F-BMD in different subgroups stratified according to the following variables in the ovariectomized model group: different animal species, different laboratory drugs, and the duration of treatment. In the subgroup analysis of these factors, the mice group showed better effect size than the rat group (SMD = 5.55 ± 2.64 versus SMD = 1.93 ± 0.37, *P* = 0.008, Figure 10(b)) with significantly reduced heterogeneity of both groups. Another subgroup analysis indicated that the effect of Q derivatives was better than Q with better evaluation effect size (SMD = 3.28 ± 0.78 versus SMD = 1.64 ± 0.41, *P* = 0.0003, Figure 10(c)), and the heterogeneity experienced a marked decline in Q derivatives treatment group. In addition, the shorter period of Q or its derivatives treatment showed batter effect size than the longer treatment (SMD = 5.55 ± 2.64 versus SMD = 1.69 ± 0.43 versus SMD = 2.16 ± 0.64, *P* = 0.01, Figure 10(d)).

## 4. Discussion

### 4.1. Summary of Evidence

The first-ever preclinical systematic review included a batch of studies of acceptable quality to estimate the efficacy and mechanisms of Q and its derivatives in animal models of osteoporosis. The findings revealed Q and its derivatives are potential antiosteoporosis drug via multiple mechanisms.

### 4.2. Limitations

Some limitations of the meta-analysis and the system evaluation were listed as follows: (1) there may still be a certain degree of selective bias due to the lack of negative studies and the studies from other databases or in other languages; (2) defects in aspects of blinding assessment of outcome and sample size calculation may affect the accuracy of findings [47]; (3) the few number of studies modeled by nonovariectomized methods leads to that only comparisons between groups are carried out rather than systematic evaluations in those studies; (4) no study utilized animals with relevant complication.

### 4.3. Implication

#### 4.3.1. Animal Model Selection

Using different animal models at different research stages of disease is crucial to study it pathophysiology and treatments [48]. Factors that need to be considered include pathogenesis of model, availability of the animals, technical requirements, and cost and ethical considerations [49]. According to the pathogenesis, animal models of osteoporosis can be divided into two types: models with increased bone resorption as the dominant mechanism (such as ovariectomized osteoporosis model, disused osteoporosis model, retinoic acid induction model, nutritional osteoporosis model, and glucocorticoid model) and models with reduced bone formation as the dominant mechanism (such as senile osteoporosis model and n-ZnO induction model) [50]. The present study comprehensively contains the ovariectomized osteoporosis model, retinoic acid or n-ZnO induction model, diabetic osteoporosis, and glucocorticoid model to estimate the efficacy and mechanisms of Q and its derivatives for osteoporosis. And the results of meta-analysis and subgroup analysis reflected that Q and its derivatives could play a role in both two mechanisms of osteoporosis (SMD = 2.00 ± 0.36 versus SMD = 1.93 ± 0.58, *P* = 0.85, Figure 10(a)). However, some key points to establish incorporating models still deserve attention. The ovariectomized rats, as the best recognized postmenopausal osteoporosis model, are the most adopted model in eligible studies. Animals with mature skeleton are obligatory for osteoporosis researches [51]. In the present study, some immature rats (<12 weeks) whose bone mass was below its peak [32, 33, 43] were used to establish models, causing that the confounding factors are introduced to animals that are still accruing bone [51]. Thus, the application of animals with mature bone needs to be emphasized for future osteoporosis experiments. In addition, the dosage of glucocorticoids should be grasped well to avoid no change in bone mass in low dosage or animal death due to overimmunization in high dosage. Preexperiment as Derakhshanian et al. [41] did to explore the appropriate dosage is a recommended practice.

The subgroup analysis showed better effect size in the mice group than the rat group (SMD = 5.55 ± 2.64 versus SMD = 1.93 ± 0.37, *P* = 0.008, Figure 10(b)) with significantly reduced heterogeneity of both groups, suggesting that different animals may be one of the main sources of heterogeneity. The commonly used animals in osteoporosis experiments are rodent, rabbit, dog, sheep, primates, and so on [52, 53]. Rodents such as rats and mice possess the advantages of being repeatable, cheap, and convenient to be bred and anesthetized. It also takes less time to form a new balance of bone remodeling. After ovariectomy in rodents, the bone mass of cancellous bone decrease and the bone turnover rate increase, which resembles to that of osteoporosis in postmenopausal women and estrogen replacement therapy could alleviate bone mass loss [54]. Therefore, female rodents are widely used in the study of postmenopausal osteoporosis. It was regarded as a preferred animal for small- or medium-sized laboratories to investigate the efficacy and mechanisms of drugs. However, it is not suitable for the study of the bone cortex because of the absence of haversian system in the bone cortex of rats or mice. Additionally, difficulties arose when the arm of the study was to implant fixation or prosthetic devices as well as that studies which need several the collection of high blood volumes or surgical operation or several biopsies due to its small size especially in mice [55]. Compared to rodents, rabbits and dogs have the convenience of appropriate cost in terms of purchase and maintenance and reasonable anatomical size which was advantageous to biopsies, blood collection repeatedly, and surgical treatments such as bone implant and bone-implant interface. On the other hand, it is suitable for the study of the effect of cortical bone because of the obvious Harvard reconstruction. However, no significant change was observed in bone mineral density after ovariectomy in rabbits and dogs [56]. The combination of surgery and subsequent glucocorticoid treatment is the best solution at present to obtain bone mineral reduction consistently in the short term in rabbits [48]. However, it cannot be achieved in dogs now which may be related to the low estrogen level in female dogs. Apart from the advantages of rabbits and dogs, sheep are ideal models for the study of vertebral osteoporosis [57] and they also have similar toxic effects on osteocytes by fluoride to humans [58]. Unfortunately, they are not suitable to be adopted to study the efficacy of oral administration of drugs given that the sheep are ruminants. Additionally, bone mineral density, blood biochemical parameters, and bone histomorphology of sheep all fluctuated seasonally [59] which may influence the accuracy of experimental results. From both a physiological and anatomical standpoint, the characteristics of the skeleton of primates are most close to humans than any other type of animal. Nevertheless, obtaining licenses to use them as experimental animal become increasingly difficult due to ethical considerations [60] and potential epidemic animal-borne diseases in primates [61]. Meanwhile, the high cost to purchase and maintain primates restricts their use in experimentation [49]. In consideration of that, the arm of the present studies is to explore the preliminary effect of and mechanisms of Q in animal models of osteoporosis, thus the use of rodents is acceptable at this stage. We suggest the advanced animals (sheep, primates, etc.) or animal models (transgenic or knockout rodents, etc.) for osteoporosis should be chosen in the future on the basis of the experimental purpose and permissible conditions.

#### 4.3.2. Other Subgroup Analyses

A better evaluation effect size in a group of Q derivatives (SMD = 3.28 ± 0.78 versus SMD = 1.64 ± 0.41, *P* = 0.0003, Figure 10(c)) was indicated in subgroup analysis based on contrast of the overall effect in different experiments. Given the existence of mixed factors in different experiments, all included studies have been perused again for these studies were designed to contrast the different therapeutic effects between Q and its derivatives under the same experimental conditions. Among, QCG (quercetin-6-C-A-D-glucopyranoside) [27] and rutin (quercetin-3-O-B-rutinoside) [33] were reported to improve bone biomechanical quality more effectively than Q via positive modifications of bone microarchitecture and bone mineral density without hyperplastic effect on the uterus, which possibly is related to that the synthesis of modified groups attached to Q improves solubility and bioavailability [62]. In this context, all the existing derivatives of Q were collected and listed in Table 4, which are recommended as potential antiosteoporosis drugs in the future researches. Another subgroup analysis showed the shorter period of Q or its derivative treatment showed better effect size than the longer treatment (SMD = 5.55 ± 2.64 versus SMD = 1.69 ± 0.43 versus SMD = 2.16 ± 0.64, *P* = 0.01, Figure 10(d)), suggesting that the duration of treatment may be a source of high heterogeneity. For the reason, we attribute it to that osteoporosis is a progressive and irreversible disease when pathogeny persist, extending the treatment time of Q and its derivatives is merely conducive to delay the progression of osteoporosis rather than reverse it.

#### 4.3.3. Possible Mechanisms

**Figure 11:**
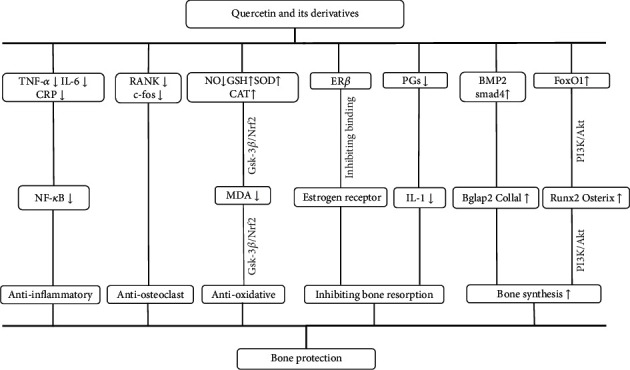
A schematic representation of osteoprotective mechanisms of Q and its derivatives for osteoporosis.

Systemic review of preclinical studies is conducive to understand comprehensively pathological mechanisms of disease and pharmacological effects of drugs [63]. We have summarized the possible mechanisms of Q and its derivatives mediated bone protection from current findings and listed them as follows: (1) Q alleviated oxidative damage by decreasing NO [36] and increasing GSH [31, 40], SOD [39, 45], and CAT [45] to reduce the release of MDA [31, 39] in the ovaries and bone tissue. A decreased MDA level in the ovaries directly increased estrogen activity [31] which has been shown to have antioxidant properties [64, 65]. In addition, Gsk-3*β*/Nrf2 signal pathway was reported to participate in the regulation of the abovementioned antioxidant process [39]. (2) Q and quercetin-3-O-rutinose have the similar effect of phytoestrogen on inhibiting bone resorption by participating in the binding of estrogen receptor (ER) [33, 45, 66], especially in ER*β* mainly expressed in bone [67]. However, another study [43] showed that Q did not appear to have this activity through either ER*α* or ER*β*, which suggested that Q might affect bone metabolism through ERs independent pathway. (3) Q alleviated inflammatory reaction by inhibiting the expression of TNF-*α* [4, 32, 36], IL-6 [4, 36], INF-*γ* [4], and CRP [36]. And then TNF-*α* activated NF-*κ*B, increased the expression of NF-*κ*B protein, and promoted the degradation of *β*-catenin protein [31]. (4) Q promoted bone synthesis by enhancing the expression of osteogenic protein (FoxO1, Bglap2, Collal, Osterix, and Runx2) [30, 39] via PI3K/Akt/Fox O1/NF-*κ*B signal pathway [30] and BMP2/smad4 signaling pathway [35]. (5) QCG inhibited the expression of osteoclast markers including RANK and c-fos in bone marrow cells (BMCs) cultured in the presence of RANK ligand and M-CSF [27]. Q and quercetin-3-O-beta-D-glucuronide inhibited RANK-induced osteoclast formation in a dose-dependent manner in RAW264.7 cells, and the RANK ligand-stimulated expression of osteoclast related genes including NFATc1 was inhibited by Q [43]. In addition, as the most abundant p38 member in the bone and bone marrow [68], the ablation of p38 MAPK signaling in osteoblast lineage cells protects the mice from bone loss induced by estrogen deficiency [34, 69] found that Q could attenuate osteoporosis by downregulating MAPK signaling pathways. (6) Prostaglandins (PGs) played a role in IL-1-induced bone resorption [70]. Q was observed to reduce the production of PGs by inhibiting cyclooxygenase and phosphoesterase A2 [71]. The mechanism diagram is summarized in Figure 11.

## 5. Conclusion

The present study provided the preliminary preclinical evidence that oral administration of Q and its derivatives was capable of partially reversing osteopenia in animal models probably via antioxidant, anti-inflammatory, promoting osteogenesis, inhibiting osteoclasts, and its estrogen-like effect. The findings reveal the possibility of developing Q and its derivatives as a drug or an ingredient in diet for the clinical treatment of osteoporosis.

## Figures and Tables

**Table 1:** Characteristics of the included studies.

Study (years)	Species (sex, n = experimental/control group, weight)	Model (method)	Anesthetic	Treatment group (method to astragal sides)	Control group	Outcome index (time)	Intergroup differences
Geng et al. 2019 [4]	Female SD rats (10/10, 340-350 g, 8-month-old)	Bilateral oophorectomy was performed on rats	Chloral hydrate	By oral gavage of quercetin-3-O-rutinose (10 mg/kg/d, qd) for 3 months after modeling	By oral gavage of NS after modeling and lasted 3 months	1. BMD (femur) 2. Uterine index 3. Survival rate 4. Serum IL-6, TNF-*α*, and INF-*γ*	1. *P* < 0.05 2. *P* < 0.05 3. *P* < 0.05 4. *P* < 0.05
Min 2019 [30]	Female SD rats (10/10, 265.70 ± 7.89 g, 8-10-week-old)	Bilateral oophorectomy was performed on rats	Pentobarbital sodium	By oral gavage of quercetin (50 mg/kg) for 8 weeks after modeling	By oral gavage of 3 ml CMC-Na after modeling and lasted 8 weeks	1. BMD (femur) 2. Serum Ca and P 3. Urinary Ca and P 4. Bone-related parameters under micro-CT (Tb.N and Tb.Th) 5. BV/TV 6. Serum ALP 7. FoxO1 and NF-*κ*B 8. Runx2 and Osterix	1. *P* < 0.05 2. *P* > 0.05 3. *P* < 0.05 4. *P* < 0.05 5. *P* < 0.05 6. *P* < 0.05 7. *P* < 0.05 8. *P* < 0.05
Nada et al. 2018 [31]	Female Y59 rats (10/10, 200–250 g, 3-month-old)	By oral gavage of isotretinoin (80 mg/kg, qd) for 14 days	A mixture of ketamine and xylazine (10 mg/kg)	By oral gavage of quercetin (100 mg/kg, qd) after modeling and lasted 2 weeks	By oral gavage of isometric physiological solution with 0.5% ethanol after modeling and lasted 2 weeks	1. Bone pathology 2. BMD (femur) 3. Bone weight index 4. Uterine weight index 5. Serum ALP and LDH 6. Serum AST, ALT, TP, GLU, and BUN 7. Serum GSH, SOD, MDA, and CAT 8. Haematological parameters 9. The length and the diameter of the femur bone	1. *P* < 0.05 2. *P* > 0.05 3. *P* < 0.05 4. *P* < 0.05 5. *P* > 0.05 6. *P* < 0.05 7. *P* < 0.05 8. *P* < 0.05 9. *P* < 0.05
Yuan et al. 2018 [32]	Female SD rats (10/10, 265.70 ± 7.89 g, 8-12-week-old)	Bilateral oophorectomy was performed on rats under general anaesthesia with an abdominal longitudinal incision	NM	By oral gavage of quercetin (50 mg/kg, qd) after modeling and lasted 8 weeks	By oral gavage of 3 ml CMC after modeling and lasted 8 weeks	1. BMD (femur) 2. Maximum load, radialis elasticity, elastic load 3. Bone-related parameters under micro-CT (Tb.N and Tb.Th) 4. Serum TNF-*α* 5. NF-*κ*B	1. *P* < 0.05 2. *P* < 0.05 3. *P* < 0.05 4. *P* < 0.05 5. *P* < 0.05
Yuan et al. 2018 [33]	Female SD rats (9/7, 230-280 g, 10-11-week-old)	Bilateral oophorectomy was performed on rats under general anaesthesia with a median incision of back	NM	By oral gavage of quercetin (50 mg/kg, qd) after modeling and lasted 12 weeks	By oral gavage of 3 ml CMC after modeling and lasted 12 weeks	1. BMD (femur) 2. Survival rate 3. Bone-related parameters under micro-CT (Tb.N and Tb.Th, BV/TV, SMI) 4. Uterine weight 5. Urinary Ca and P 6. Serum ALP	1. *P* < 0.01 2. *P* > 0.05 3. *P* < 0.05 4. *P* < 0.05 5. *P* > 0.05 6. *P* < 0.05
Yuan et al. 2018 [33]	Female SD rats (9/7, 230-280 g, 10-11-week-old)	Bilateral oophorectomy was performed on rats under general anaesthesia with a median incision of back	NM	By oral gavage of quercetin-3-O-rutinose (50 mg/kg, qd) after modeling and lasted 12 weeks	By oral gavage of 3 ml CMC after modeling and lasted 12 weeks	1. BMD (femur) 2. Survival rate 3. Bone-related parameters under micro-CT (Tb.N and Tb.Th, BV/TV, SMI) 4. Uterine weight 5. Urinary Ca and P 6. Serum ALP	1. *P* < 0.01 2. *P* > 0.05 3. *P* < 0.05 4. *P* < 0.05 5. *P* > 0.05 6. *P* < 0.05
Xing et al. 2017 [34]	Female SD rats (10/10, 220-240 g, 6-month-old)	Bilateral oophorectomy was performed on rats under general anaesthesia with a median incision of back	Ketamine	By oral gavage of quercetin (200 mg/kg, qd) after modeling and lasted 60 days	By oral gavage of isometric H_2_O after modeling and lasted 60 days	1. BMD (femur) 2. Serum CTX, TRAP, PINP, and Runx2 3. Maximum load 4. Serum BUN, SCr, ALP, type 1 procollagen, Ca, and P 5. P-ERK, ERK, P-JNK, JNK, P-P38, P38	1. *P* < 0.05 2. *P* < 0.05 3. *P* < 0.05 4. *P* < 0.05 5. *P* < 0.05
Zheng et al. 2017 [35]	Female SD rats (10/10, 190-210 g, 3-month-old)	Bilateral oophorectomy was performed on rats under general anaesthesia	Chloral hydrate (1 ml/100 g)	By oral gavage of quercetin (200 mg/kg, qd) after modeling and lasted 60 days	By oral gavage of isometric NS after modeling and lasted 60 days	1. BMD (lumbar) 2. Serum estradiol, ALP, OC, PINP, TRACP-5b, CTX, Ca, and P 3. Serum Ca and P, urinary Ca, and P 4. BMP2 and Smad4	1. *P* < 0.01 2. *P* < 0.05 3. *P* < 0.01 4. *P* < 0.01
Abdelkarem et al. 2016 [36]	Male Wistar albino rats (10/10, 170-200 g, NM)	By oral gavage of n-ZnO (600 mg/kg, qd) for 5 consecutive days	Ether	By oral gavage of quercetin (200 mg/kg, qd) after modeling and lasted 3 weeks	By oral gavage of nothing after modeling and lasted 3 weeks	1. Serum ALP and CTX 2. Serum NO 3. DNA level in liver tissues 4. Serum TNF-*α*, IL-6, and CRP 5. Serum Ca, P, and Mg	1. *P* < 0.05 2. *P* < 0.05 3. *P* < 0.05 4. *P* < 0.05 5. *P* < 0.05
Bian et al. 2016 [37]	Female SD rats (10/10, 190-210 g, 3-month-old)	Bilateral oophorectomy was performed on rats under general anaesthesia with a median incision of back	Chloral hydrate (1 ml/1 kg)	By oral gavage of quercetin (200 mg/kg, qd) after modeling and lasted 12 weeks	By oral gavage of isometric NS after modeling and lasted 12 weeks	1. Serum estradiol, OC 2. The 3-point bending test	1. *P* < 0.05 2. *P* < 0.05
Feng et al. 2016 [38]	Female SD rats (10/10, 250-310 g, 6-month-old)	Bilateral oophorectomy was performed on rats under general anaesthesia with an abdominal longitudinal incision	Sodium pentobarbital	By oral gavage of quercetin (200 mg/kg, qd) after modeling and lasted 3 months	By oral gavage of isometric NS after modeling and lasted 3 months	1. Bone pathology 2. Survival rate 3. Serum OC and type I collagen protein	1. *P* < 0.05 2. *P* < 0.05 3. *P* < 0.05
Zhou 2016 [39]	Male C57BL/6 mice (20/20, 18.23 ± 0.56 g, 4-week-old)	Feeded with a high-fat diet (45% of energy comes from fat) for 17 weeks	Ethyl ether	Feeded with a high-fat diet+0.01% quercetin for 17 weeks	Feeded with a high-fat diet for 17 weeks	1. BMD (femur) 2. Bone length and diameter 3. Femoral Ca and P 4. Maximum load 5. Femur weight 6. Serum SOD, MDA, T-AOC, and GSH 7. Serum Runx2, col1a1, Bglap2, RANKL, CTSK Nrf2, TR*α*1, and GSK-3*β*	1. *P* < 0.05 2. *P* < 0.05 3. *P* < 0.05 4. *P* < 0.05 5. *P* < 0.05 6. *P* < 0.05 7. *P* < 0.05
Tian et al. 2014 [40]	Female C57BL/6 mice (20/20, 17.27 ± 0.71 g, 4-week-old)	Feeded with a high-fat diet (20% of energy comes from fat) for 26 weeks	NM	Feeded with a high-fat diet+0.01% quercetin for 26 weeks	Feeded with a high-fat diet for 26 weeks	1. Maximum load 2. Bone length and diameter 3. Femur weight 4. Femoral Ca and mineral 5. Serum GSH, GSSG, and MDA	1. *P* < 0.05 2. *P* < 0.05 3. *P* < 0.05 4. *P* < 0.05
Derakhshanian et al. 2012 [41]	Female SD rats (8/8, 180-240 g, 6-7-month-old)	By subcutaneous injection of methylprednisolone sodium succinate (40 mg/kg body mass) for 6 weeks	A mixture of ketamine (50 mg/kg) and xylazine (30 mg/kg)	By oral gavage of quercetin (150 mg/kg, tiw) after modeling and lasted 6 weeks	By oral gavage of isometric CMC after modeling and lasted 6 weeks	1. Bone pathology 2. Maximal load 3. The 3-point bending test 4. Serum OC and CTX 5. Serum Ca and P	1. *P* < 0.05 2. *P* < 0.05 3. *P* < 0.05 4. *P* < 0.05 5. *P* < 0.05
Liang et al. 2011 [42]	Male SD rats (10/10, 200-220 g, NM)	By intraperitoneal injection of STZ (100 mg/kg)	Diethyl ether	By oral gavage of quercetin (50 mg/kg, qd) after STZ injection and lasted 8 weeks	By oral gavage of isometric NS after STZ injection and lasted 8 weeks	1. BMD (femur) and BMC 2. Urinary DPD and Cr 3. Serum OC and ALP 4. Bone-related parameters under micro-CT (the trabecular bone mass and microarchitecture, MAR, BFR/BS, MS/BS, and Oc.S/BS) 5. The 3-point bending test 6. Serum SOD, GSH, GST, and CAT	1. *P* < 0.05 2. *P* < 0.05 3. *P* < 0.05 4. *P* < 0.05 5. *P* < 0.05 6. *P* < 0.05
Siddiqui et al. 2011 [27]	Female SD rats (10/10, 180-200 g, NM)	Bilateral oophorectomy was performed on rats	NM	By oral gavage of quercetin (5 mg/kg, qd) after modeling and lasted 12 weeks	By oral gavage of isometric NS after modeling and lasted 12 weeks	1. BMD (femur) 2. Uterine weight 3. Bone-related parameters under micro-CT (the trabecular bone volume, separation, Tb.N, and Tb.Th) 4. RANK, M-CSF, and C-FOS	1. *P* < 0.05 2. *P* < 0.05 3. *P* < 0.05 4. *P* < 0.05
Siddiqui et al. 2011 [27]	Female SD rats (10/10, 180-200 g, NM)	Bilateral oophorectomy was performed on rats	NM	By oral gavage of QCG (5 mg/kg, qd) after modeling and lasted 12 weeks	By oral gavage of isometric NS after modeling and lasted 12 weeks	1. BMD (femur) 2. Maximal load, stiffness, and energy of femur 3. Uterine weight 4. Bone-related parameters under micro-CT (the trabecular bone volume, separation, Tb.N, and Tb.Th) 5. RANK, M-CSF, and cFOS	1. *P* < 0.05 2. *P* < 0.05 3. *P* < 0.05 4. *P* < 0.05 5. *P* < 0.05
Tsuji et al. 2009 [43]	Female C57BL/6J mice (7/7, NM, 9-week-old)	Bilateral oophorectomy was performed on mice	NM	Feeded with the control diet +2.5% quercetin (5 g per mouse, qd) after modeling and lasted 4 weeks	Feeded with the control diet (5 g per mouse, qd) after modeling and lasted 4 weeks	1. BMD (lumbar and femur) 2. Uterine weight 3. Femoral bone parameters measured by pQCT (cortical area and thickness) 4. Bone-related parameters under a semiautomated system (BV/TV, Tb.N, Tb.Th, Tb.Sp, OV/BV, OS/BS, O.Th, Ob.S/BS, ES/BS) 5. CTR, CTSK, MMP9, and NFATc1	1. *P* < 0.05 2. *P* < 0.05 3. *P* < 0.05 4. *P* < 0.05 5. *P* < 0.05
Wang et al. 2008 [44]	Female SD rats (8/8, 200-250 g, 3-month-old)	Bilateral oophorectomy was performed on rats under general anaesthesia	Ketamine (60 mg/kg)	By oral gavage of quercetin (300 mg/kg, qd) in 1 week after modeling and lasted 16 weeks	By oral gavage of isometric NS in 1 week after modeling and lasted 16 weeks	1. BMD (lumbar and femur) 2. Observation of vaginal smear in rats 3. Uterine weight index 4. Uterine pathology	1. *P* < 0.05 2. *P* < 0.05 3. *P* < 0.05 4. *P* < 0.05
Zhu and Wei 2005 [45]	Female Wistar rats (10/10, 241 ± 24 g, 3-month-old)	Bilateral oophorectomy was performed on rats with an abdominal longitudinal incision	NM	By oral gavage of quercetin (200 mg/kg, qd) after modeling and lasted 3 months	By oral gavage of 3 ml NS after modeling and lasted 3 months	1. BMD (femur) 2. BMC 3. The 3-point bending test 4. Survival rate 5. Serum estradiol, ALP, ACP	1. *P* < 0.05 2. *P* < 0.05 3. *P* < 0.05 4. *P* < 0.05 5. *P* < 0.05
Marie 2000	Female Wistar rats (10/10, 206 ± 5 g, 3-month-old)	Bilateral oophorectomy was performed on rats	Chloral hydrate	Feeded with the control diet + quercetin-3-O-rutinose (2.5 g/kg diet, qd) for 90 days after modeling	Feeded with the control diet for 90 days after modeling	1. BMD (femur) 2. Serum OC 3. Serum Ca and urinary Ca	1. *P* < 0.05 2. *P* < 0.05 3. *P* < 0.05

Note: QCG: quercetin-6-C-A-D-glucopyranoside; BMD: bone mineral density; ALP: alkaline phosphatase; LDH: lactate dehydrogenase; AST: aspartate aminotransferase; ALT: alanine aminotransferase; TP: total protein; Glu: glucose; BUN: blood urea nitrogen; GSH: glutathione peroxidase; SOD: superoxide dismutase; MDA: malondialdehyde; CAT: catalase; CMC: carboxymethyl cellulose; SD rats: Sprague Dawley rats; TNF-*α*: tumor necrosis factor-*α*; NF-*κ*B: nuclear factor-*κ* B; Tb.N: trabecular linear density; Tb.Th: trabecular thickness; BV/TV: object surface/volume ratio; SMI: structure model index; OC: osteocalcin; CTX: C-terminal cross-linked telopeptide of type I collagen; TRAP: tartrate resistant acid phosphatase; SCr: serum creatinine; PINP: N-terminal propeptide of type 1 procollagen; TRACP-5b: tartrate-resistant acid phosphatase 5b; BMP2: bone morphogenetic protein 2; Smad4: Smad family member 4; Runx2: runt-related transcription factor 2; NS: normal saline; CTSK: cathepsin K; Bglap2: bone Gla protein 2; CRP: C-reactive protein; RANKL: receptor activator of nuclear factor-*κ* B ligand; Nrf2: NF-E2-related factor 2; TR*α*1: thyroid hormone receptor *α*1; GSK-3*β*: glycogen synthase kinase 3*β*; GSSG: oxidized glutathione; STZ: Streptozotocin; T-AOC: total antioxidative capacity; DPD: deoxypyridinoline; BMC: bone mineral content; MAR: mineral apposition rate; BFR/BS: bone formation rate per bone surface; MS/BS: mineralizing surface per bone surface; Oc.S/BS: osteoclast surface per bone surface; GST: glutathione S-transferase; M-CSF: macrophage colony-stimulating factor; pQCT: peripheral quantitative computed tomography; Tb.Sp: trabecular separation; OV/BV: osteoid volume per bone volume; OS/BS: osteoid surface per bone surface; O.Th: osteoid thickness; ES/BS: eroded surface per bone surface; CTR: calcitonin receptor; MMP9: matrix metalloproteinase 9; NFATc1: nuclear factor of activated T cells c1; ACP: acid phosphate.

**Table 2:** Information on quercetin or its derivatives of each study.

Study (years)	Chemical composition	Source	Purity (%)	Quality control reported
Min et al. 2019 [30]	Quercetin	Sigma-Aldrich Corporation, USA	(≥99%)	Batch number: XSD201510008, HPLC
Geng et al. 2019 [4]	Quercetin-3-O-rutinose	National Institute of controlled drugs and biological products, China	(≥98%)	HPLC
Nada et al. 2018 [31]	Quercetin	Aldrich Ch. Co. Inc. Milwaukee WI, USA	(98%)	?
Yuan et al. 2018 [32]	Quercetin	Sigma-Aldrich Corporation, USA	(≥99%)	HPLC
Yuan et al. 2018 [33]	Quercetin	Sigma-Aldrich Corporation, USA	(≥99%)	Batch number: XSD201510008, HPLC
Yuan et al. 2018 [33]	Quercetin-3-O-rutinose	Sigma-Aldrich Corporation, USA	(≥99%)	Batch number: XSD201510008, HPLC
Xing et al. 2017 [34]	Quercetin	?	?	?
Zheng et al. 2017 [35]	Quercetin	China Institute of Food and Drug Verification	(≥98%)	Batch number: 100081201509
Abdelkarem et al. 2016 [36]	Quercetin	Sigma-Aldrich Corporation, USA	(≥99%)	HPLC
Bian et al. 2016 [37]	Quercetin	Ai Ke Da Chemical Reagent Co., Ltd., CHN	?	HPLC
Feng et al. 2016 [38]	Quercetin	Institute of occupational health and occupational disease, Chinese Academy of Preventive Medicine, CHN	?	Batch number: 911015
Zhou et al. 2016 [39]	Quercetin	Sigma-Aldrich Corporation, USA	(≥99%)	HPLC
Tian et al. 2016 [40]	Quercetin	Sigma-Aldrich Corporation, USA	(≥99%)	HPLC
Derakhshanian et al. 2012 [41]	Quercetin	Sigma-Aldrich Corporation, USA	95%	HPLC
Liang et al. 2011 [42]	Quercetin	Sigma-Aldrich Corporation, USA	?	HPLC
Siddiqui et al. 2011 [27]	Quercetin	Sigma-Aldrich Corporation, USA	?	HPLC
Siddiqui et al. 2011 [27]	Quercetin-6-C-A-D-glucopyranoside	Purificated by themself	?	HPLC
Tsuji et al. 2009 [43]	Quercetin	Sigma-Aldrich Corporation, USA	?	HPLC
Wang et al. 2008 [44]	Quercetin	Shaanxi Huike Biology Co., Ltd., CHN	?	?
Zhu and Wei 2005 [45]	Quercetin	Products of labor and health institution, Chinese Academy of Preventive Medicine, CHN	?	Batch number: 911015
Marie 2000	Quercetin-3-O-rutinose	Sigma-Aldrich Corporation, USA	?	HPLC

HPLC: high-performance liquid chromatography.

**Table 3:** Risk of bias of the included studies.

Study	A	B	C	D	E	F	G	H	I	J	Total
Geng et al. 2019 [4]	**√**	**√**	**√**	**√**		**√**			**√**		6
Min et al. 2019 [30]		**√**	**√**			**√**			**√**		4
Nada et al. 2018 [31]	**√**	**√**		**√**		**√**			**√**	**√**	6
Yuan et al. 2018 [32]	**√**	**√**	**√**						**√**	**√**	5
Yuan et al. 2018 [33]	**√**		**√**						**√**	**√**	4
Xing et al. 2017 [34]	**√**	**√**	**√**			**√**			**√**		5
Zheng et al. 2017 [35]	**√**	**√**	**√**	**√**		**√**			**√**	**√**	7
Abdelkarem et al. 2016 [36]	**√**	**√**	**√**	**√**		**√**			**√**	**√**	7
Bian et al. 2016 [37]	**√**	**√**	**√**		**?**	**√**			**√**	**√**	6
Feng et al. 2016 [38]	**√**	**√**	**√**			**√**				**√**	5
Zhou et al. 2016 [39]		**√**	**√**			**√**			**√**		4
Tian et al. 2014 [40]	**√**	**√**	**√**						**√**	**√**	5
Derakhshanian et al. 2013 [41]	**√**	**√**	**√**			**√**			**√**	**√**	6
Liang et al. 2011 [42]	**√**	**√**	**√**			**√**				**√**	5
Siddiqui et al. 2011 [27]	**√**	**√**	**√**							**√**	4
Tsuji et al. 2009 [43]	**√**	**√**	**√**	**√**		**?**			**√**	**√**	6
Wang et al. 2008 [44]	**√**		**√**			**√**			**√**		4
Zhu and Wei 2005 [45]	**√**		**√**						**√**		3
Marie 2000	**√**	**√**				**√**			**√**		4

Note: studies fulfilling the criteria of: A: peer-reviewed publication; B: control of temperature; C: random allocation to treatment or control; D: blinded induction of model (group randomly after modeling); E: blinded assessment of outcome; F: use of anesthetic without significant protective and toxic effects on bones; G: appropriate animal model (aged, hyperlipemia, hypertensive, or diabetes); H: sample size calculation; I: compliance with animal welfare regulations (including three or more of the following points: preoperative anaesthesia, postoperative analgesia, nutrition, disinfection, environment temperature, environment humidity, circadian rhythm, and euthanasia); J: statement of potential conflict of interests.

**Table 4:** The derivatives of Q.

Classification	Name	Structural formula	Reference
Water-soluble quercetin derivatives	Sodium quercetin monosulfate	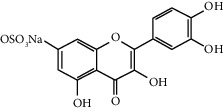	Yu 1998
	Quercetin disodium bisulfate	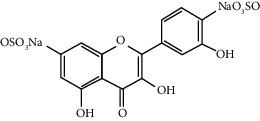	Yu 1998
	Quercetin-7-sodium sulfate	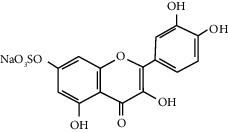	Wu 2009
	7-O-aliphatic aminoalkyl quercetin derivative	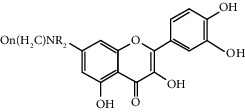	Liu 2001
	4′-aliphatic aminoalkyl substituted quercetin derivative	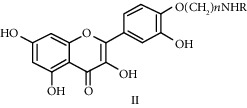	Sun 2003
	Quercetin-3′-*α*-amino acid ester hydrochloride	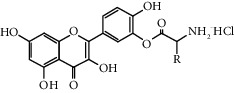	Yu 2008
	8-morpholinecyclomethyl-quercetin	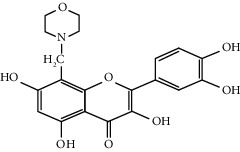	Dai 2006
	8-methylpiperazine methylcyclo-quercetin	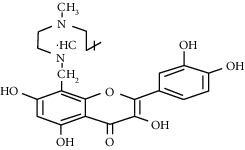	Dai 2006
	8-ethyl piperazine cyclomethyl-sheepskin	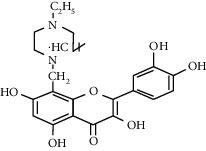	Dai 2006
	3′-O-N-carboxymethylformamide-based quercetin	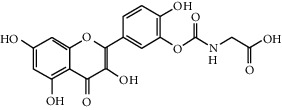	Golding 1997

Liposoluble quercetin derivatives	Quercetin-6-C-A-D-glucopyranoside	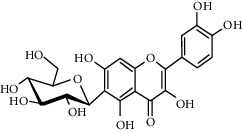	Jawed 2011
	Quercetin-3-O-rutinose	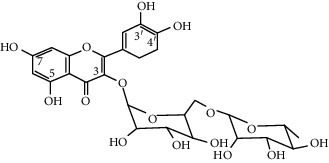	Marie 2000
	3-O-alkyltrihydroxyethyl quercetin derivative	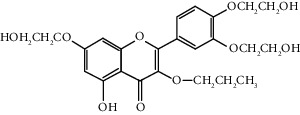	Xu 2013
	3-O-*α*-propionate trishydroxyethyl quercetin derivative	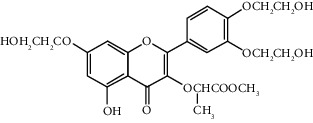	Zhao 2014
	3-O-*α*-butyrate-trishydroxyethyl quercetin derivative	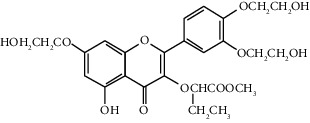	Zhao 2014
	3-O-methyl-quercetin	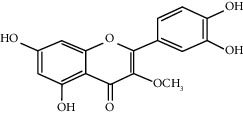	Li 2004
	Trishydroxyethyl quercetin	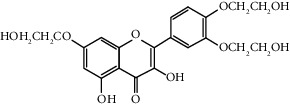	Xu 2013
	Quercetin-3-O-*β*-D-glucuronide	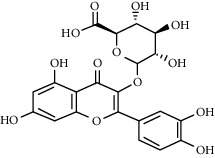	Mitsuyoshi 2009
